# Health Resorts and Mineral Waters

**Published:** 1893-08

**Authors:** Robert Newman

**Affiliations:** New York City


### HEALTH RESORTS AND MINERAL WATERS.

"Mama, when are we going to the country?" is the query of almost every child when the summer vacation in the schools draws near.

Going to the country has become fashionable; every office boy wants his vacation, and many servants leave good situations for the sake of going to a favorite resort, and in engaging make it a *sine qua non* that the family must take her to Newport. At the present time a general stampede has taken place to Chicago, from which many a servant will return in due time, a wiser woman with "experience." Mothers generally think they must take their children to the country. The question now arises, is it really so necessary to emigrate in summer? In a general way, I say no; it is only requisite to live naturally in a healthy air and surroundings. Man can live any where in the world, and will submit to the most diverse conditions imposed by nature or man. To do so depends on his state of health and training, so that he has stored up in his system "energy," which can be drawn upon to meet the vicissitudes of his changing environment.

Health and mental vigor will be best secured by the exercise and co-ordination of large numbers of reflex areas in widely separated parts of the body.

But if I were asked, "Is a change of air and surroundings pleasant and beneficial?" I would say decidedly, "Yes"—and for many even a necessity. While I condemn the fashion or abuse of running to the country, I favor the change for rest and recreation, and consider a health resort a necessity for sick or overworked bodies.

We live in America, as a rule, in extremes; there is either a standstill or a rush, which causes overwork of mind and body in one case, or depression and worry in the other. Many have no idea how our clergy work. There is every day a morning and evening service, lots of calls on the sick and well, attendance at births, weddings and funerals, presiding over the branch societies, guilds, clubs, and kindred organizations, looking after the poor, insane, deaf-mutes and cripples, arranging meetings for extra occasions, holidays, service of Sunday schools, besides the regular service and sermons for Sunday. Now, what time is left them to look after their own families? Such a minister needs rest for body and mind, and his congregation generally sends him to the country or foreign lands for recuperation.

And after all, such vacations are no innovation; on the contrary, they were known among the ancients. Hippocrates considered change of residence *pro tem.* as beneficial; Celsus was a climatologist, and advised journeys to the country and ocean; Plivius claimed benefit for consumptives among the pine forests; Galen sent patients to the mountains; Laennec was an enthusiast about sea air, and himself died in a room the floor of which was covered with sea grass.

Dr. H. Brehmer was the first who established a sanitarium for treatment of pulmonary diseases, in Goerbersdorf.

Health resorts came in existence through public favor, people for some reason flocking together at certain places. Situations are generally for certain reasons, as climatology, elevation, scenery, accessibility, healthy surroundings, water for drinking and bathing in all varieties, pleasures in and out-doors, good hunting or fishing, or for the ease and rapidity with which they can be reached. Then, again, these resorts sometimes result from the earnest and careful thought of Mama, who by early experience knows in what kind of grounds young men are accustomed to spend their time in hunting for "deers," I presume.

If we classify such health resorts, we find

1. Ocean resorts, where people go for the sea water and air.

2. Mountain resorts, for high elevation and air accordingly.

3. Inland places, for protection against rough winds, places for solid rest and some sports, in which the lake regions are included.

4. Among the pines, which are recommended for diseases of the lungs.

5. Springs, whose waters have medicinal qualities, for certain diseases.

Wherever either of these qualities existed, health resorts have been established, and they are managed either as

1. Fashionable resorts, as Newport, mostly for pleasure-seekers;

2. Sanitarium, Battle Creek, Mich., for the afflicted; or,

3. Mixed, in which the former two are combined, such as Richfield Springs.

The management of such resorts is very different. In some the regulations are too strict even for a sick man, while in others the want of regulations and systematic diet is a great fault, and the sick are not benefited. So we find that some of our best places, though medically managed, are managed very badly, or, we may say, not managed at all.

We also find winter and summer health resorts. The tropics are naturally winter resorts, while ocean and mountain scenery belong to the summer resorts. The most successful winter resort in Jersey is Lakewood; it has a mild temperature, pure air, and is among the pines; while Atlantic City is frequented both in summer and winter.

### WHY DO PEOPLE GO TO HEALTH RESORTS?

Reasons are manifold. The majority go for pleasure and fashion, some for rest and recuperation, the minority for health. One lady came to my office and said: "Doctor, what disease must I have in order to go to Saratoga? Now please prescribe for me, and tell my husband it is absolutely necessary for me to go."

In Richfield Springs, a young lady, who was passionately fond of dancing, told me that she would not have such good chance if it was not for the beastly sulphur spring, in which her old aunt believed; and Sharon Springs is frequented by numerous high political officials, who go to the place and drink large quantities of lager, and return to the city without ever having seen the springs.

Pater familias inquires for the best watering place, where his daughters have a chance to—bathe—to make some acquaintances; you see, they are all over twenty-two.

### WATER.

But no matter where health resorts are, nearly all were established because of the presence of some water, which is really the most important factor, from which we may perhaps except mountain regions, and even there water ought to be for different uses. The importance of water cannot be over-rated; we need it everywhere and for everything. Where would our navy be without water? And a glass of good water is most refreshing, particularly if we have had enough of other liquids.

And every body knows that water is not alike everywhere. On the contrary, it differs very much in many respects, as in color, taste and composition. Every good housewife knows the difference between hard and soft water, and selects one for drinking and cooking and the other for washing clothes.

We find differences in the springs, rivers, lakes and oceans; colors vary from a transparent color to brown, blue, green in all different shades; water may be pure, or have different additions which will act differently and may make it medicinal; and you all know that there exist even hot springs and burning springs. The purest water is not always the best; on the contrary, as a drink, it is very insipid. Pure water we receive often on board of steamers, when sea water has been condensed by steam.

*Water* originally is composed of

Hydrogen, - 2 parts.
Oxygen, - - 1 part.

This composition of water was first demonstrated by Mr. Cavendish, 1781. We have two methods of research in chemistry:

1. *Analytic*, in which the compound is resolved into its elements.
2. *Synthetical*, in which the elements are made to unite and produce the compound.

By electricity we can analyze water according to the first method only, which was done first by Nicholson in 1800, and later by Faraday. This method is called electrolysis, which can be better demonstrated if the water contains salts in solution.

The positive pole attracts oxygen and acids. (Compound liberated.)

The negative, hydrogen and alkalies. (Base of the salt.)

If the water in a *spring* contains gaseous, mineral or other constituents in quantities, different from ordinary water, sufficient to find therein ingredients of the materia medica used for medicinal purposes, we have *mineral water*.

In the different mineral waters we find principally *alkalies* and their salts, as follows:

*Carbonates*—Carbonate of sodium.
" manganese.
" of calcium.
" of magnesium
" of lithia.
*Chlorides*—Chloride of sodium.
*Sulphates*—Sulphate of sodium.
*Phosphates*—Phosphate of calcium.
*Silicates*.

Other constituents are,

*Iron*—Oxyd of iron.
Malate of iron.
Carbonate of iron.
Sulphate of iron.

*Iodine*—Bromine.

*Therapeutically*, we divide mineral waters principally into,

I.—Alkaline waters.
II.—Salines.
III.—Earthy waters (calcium).
IV.—Bitter waters (purgative).
V.—Sulphur waters.
VI.—Gaseous (carbonated).
VII.—Ferruginous.
VIII.—Arsenical.

Each person will derive the best benefit, sick or well, if a watering place is selected for him to suit his individuality. It is not the water he drinks, the baths he takes, which will invigorate him or cure his disease. He derives from it only a partial benefit. The important factors are, change of scene, rest of mind and body, relief from the cares at home, no worry of business, or notes coming due, besides the diet and regulations of the medical adviser. There is no doubt that an individual afflicted with a particular disease will fare best to go to a certain watering place selected for him by his celebrated medical professor. Now, it is very well, and apparently scientific, for the professor to tell his gouty patient to go to Carlsbad; but the poor patient who has no means to pay for such a luxury, and ekes out a miserable existence at home, looks at the professional advice in a different light, and will not follow the advice for obvious reasons.

What to do in such a case is very simple. If the mountain will not go to Mohamet, it follows Mohamet must go to the mountain. Hence, our patient must drink the best mineral water for his case at home, either the genuine, or even a good artificial mineral water. The question arises, how to find or select the best mineral water suitable to his particular case. And there is the difficulty. We find many hundreds of advertisements everywhere, praising a special mineral water, which cures all. Circulars are distributed, with the analysis of a celebrated chemist; certificates of physicians east and west are attached, to bewilder the patient. Every few days I receive circulars, samples of a new mineral water, and in most cases, attached to the same, certificates from medical authorities, and immediately following these comes the smooth-talking representative of the agent, or the proprietor himself, to demonstrate the virtues of a particular mineral water.

Now, it is granted that many springs have certain medicinal qualities, more or less, while many, many more are perfectly useless, and the best that can be said about them is, they may be harmless. How can a patient, or even a doctor, select now the proper mineral water ?

It is an unfortunate fact that in America goods are selected known best to favor, and become popular for no other reason than they are advertised largely. Hence, the name of an indifferent mineral water, well advertised, we will find on all menus, while a better article, standing on its real value and merit, but *not* advertised, is unknown to the multitude.

For certain states or diseases we have, as stated before, favorite places and mineral waters.

From experience and years of observation, my favorite mineral water is "Teplitz," because

1. It has a therapeutical action on all mucous membranes.
2. It has a specific effect on diseases of the kidneys.
3. It is a pleasant table water, which also will mix well with wines and liquors, without changing into tannic acid.

For *these* reasons I recommend "*Teplitz Mineral Water.*"

New York City. ROBERT NEWMAN, M. D.

(To be concluded.)

